# Neural correlates underlying high-frequency stimulation-induced secondary hyperalgesia in humans

**DOI:** 10.1097/PR9.0000000000001342

**Published:** 2025-10-15

**Authors:** Sophie Clarke, Vishvarani Wanigasekera, Richard Rogers, Ombretta Caspani, André Mouraux, Francesca Fardo, Nanna B. Finnerup, Rolf-Detlef Treede, Irene Tracey

**Affiliations:** a Wellcome Centre for Integrative Neuroimaging, FMRIB, Nuffield Department of Clinical Neurosciences, University of Oxford, Oxford, United Kingdom; b Department of Neurophysiology, Mannheim Center for Translational Neurosciences (MCTN), University of Heidelberg, Mannheim, Germany; c Institute of Neuroscience (IoNS), Université Catholique de Louvain (UCLouvain), Brussels, Belgium; d Danish Pain Research Center, Department of Clinical Medicine, Aarhus University, Aarhus, Denmark; e Center of Functionally Integrative Neuroscience, Department of Clinical Medicine, Aarhus University, Aarhus, Denmark

**Keywords:** High-frequency stimulation (HFS), Functional magnetic resonance imaging (fMRI), Experimental pain model, Central sensitisation, Secondary hyperalgesia, Pain

## Abstract

**Introduction:**

Central sensitisation (CS), a mechanism that contributes to chronic pain, is partly characterised by increased pain responses to noxious stimuli (hyperalgesia). High-frequency electrical stimulation (HFS) of the skin using surface electrodes is a method to induce CS. Neural correlates of CS induced by HFS are not fully elucidated.

**Objectives:**

To characterise neural correlates of HFS-induced CS in healthy humans using functional magnetic resonance imaging to measure brain activity.

**Methods:**

Eighteen healthy participants completed magnetic resonance imaging scans before and after onset of HFS-induced hyperalgesia. Scans measured the neural signal during 18 noxious punctate stimuli applied 1 cm outside the HFS site (secondary hyperalgesia area) and during rest. Whole-brain, mixed-effects analysis with correction for multiple comparisons was performed for punctate-evoked neural activity. Whole-brain seed-based functional connectivity analysis was conducted to detect HFS-induced connectivity changes, using periaqueductal grey and nucleus cuneiformis seed regions.

**Results:**

High-frequency electrical stimulation induced significant hyperalgesia during punctate stimulation accompanied by increased neural activity in areas involved in pain perception including posterior insula, mid-anterior cingulate cortex, thalamus, and nucleus cuneiformis. Negative functional connectivity between the periaqueductal grey and pain-related cortical regions (insular and secondary somatosensory cortex) was reduced by HFS.

**Conclusion:**

Neural correlates of HFS-induced CS are consistent with other experimental CS models such as capsaicin, particularly increased activity of the nucleus cuneiformis – a nucleus of the descending pain modulatory system implicated in human and animal models of CS. Furthermore, changes in functional connectivity between brain regions involved in descending pain modulation suggest a shift toward facilitation over inhibition.

## Abstract 2

High-frequency stimulation increased neural activity in pain-related brain regions and altered functional connectivity between key areas of pain modulation, as measured using functional magnetic resonance imaging.

## 1. Introduction

Central sensitisation (CS) has been shown to underpin many features of chronic nociceptive, neuropathic, or nociplastic pain.^44^ As experimentally induced CS is usually limited to approximately 1 day (corresponding to acute post-traumatic pain), it is unclear how well it reflects chronicity. Nonetheless, even in acute CS models, there is a clear interaction of CS with the descending pain modulatory system (DPMS), which plays a significant role in long-term pain regulation. Preclinical research elegantly demonstrates the role of key brainstem areas of the DPMS and modulation of their activity by analgesics.^43,45,50^ Furthermore, dysregulation or loss of control of the DPMS has been attributed to development and maintenance of chronic pain states in animal studies.^5,33^ This finding has been similarly documented in experimental human studies of CS, and it has been shown there are deficits in the DPMS in several chronic pain conditions, such as fibromyalgia and diabetic painful neuropathy,^12,23,40^ making this system highly relevant in the understanding of chronic pain and identifying therapeutic targets.

Heightened pain perception to noxious stimuli (hyperalgesia) and a painful response to non-noxious stimuli (allodynia) in adjacent uninjured areas are considered key features of CS. In vivo mechanistic studies of CS in humans have used experimental models eliciting these features. High-frequency electrical stimulation (HFS) is one such model that reliably induces hyperalgesia in the adjacent normal body area (secondary hyperalgesia) and is used by multiple research groups.^36^

The HFS model delivers electrical stimulation to the skin using a multipin electrode designed to preferentially activate cutaneous nociceptors using a validated protocol based on preclinical electrical stimulation studies that produced long-term potentiation of spinal nociceptive pathways.^18^ High-frequency electrical stimulation rapidly induces (∼15 minutes) a stable area of secondary hyperalgesia to noxious pinprick stimuli, which plateaus at 30 to 45 minutes and lasts several hours (∼6 hours, but up to several days in 1/3 of healthy subjects).^18–20,35,47^ Research has shown that specific nociceptor subtypes are involved in the induction of secondary hyperalgesia by HFS; with predominant C-fibre contribution, of which the highest contribution is from TRPV1-positive C-fibre nociceptors.^15^

Neural activity underpinning the human HFS-induced response has been studied using pinprick-evoked brain potentials with an electroencephalogram, and RIII nociceptive reflex of the lower limb.^27^,^46^ These studies showed that the HFS model enhances spinal and brain responses to noxious stimuli, similarly to the effects of topical capsaicin, another well-established model for CS.

Neuroimaging has been used for mapping brain neural changes induced by CS in human experimental models as well as their modulation by analgesics.^3,16,24,25,48,49^ In keeping with preclinical research findings, neural activity changes induced by experimental models of CS have been detected along the neuroaxis from the cortex to spinal cord including functional connectivity (Fc) changes between regions. These studies show that the periaqueductal grey (PAG), rostroventromedial medulla, and nucleus cuneiformis (NCF), key regions of the brainstem DPMS that is implicated in CS, are activated during capsaicin-induced mechanical hyperalgesia, and the NCF activity to be specific to the CS state itself rather than merely reflecting a heightened pain response.^25,29,37,55^

High-frequency electrical stimulation is an established human model of CS. With its rapid onset of secondary hyperalgesia that is stable over several hours, no ongoing sensation after initial stimulation, and standardised methodology,^18^ it is ideally placed to be developed as a biomarker for investigating analgesic effects in clinical trials during early drug development as its longer duration facilitates postinjury drug administration that better simulates the clinical situation. Unlike other experimental human models of CS, there are no imaging studies elaborating the neural correlates of the HFS model. Evidence of its neural effects is limited to evoked potential-derived electroencephalogram metrics and nociceptive reflex activity of the limbs.^27,46^

This study aimed to characterise the brain neural correlates of CS induced by HFS in healthy humans as measured by functional magnetic resonance imaging (fMRI). It was hypothesised that HFS-induced secondary mechanical hyperalgesia would increase the neural activity as measured by the blood-oxygen-level–dependent (BOLD) response to pinprick stimulation, in some brain regions previously shown to have increased activity using other experimental human models of CS. These include the insular cortex, anterior cingulate cortex (ACC), secondary somatosensory cortex (SII), thalamus, and brainstem regions; especially the NCF given this region's specific role in the maintenance of CS.^25,29,55^ Second, we hypothesised that PAG and NCF, areas implicated in the human experimental models of capsaicin-induced CS, would have altered connectivity with other brain regions.^25,32^ For seed-based Fc analysis, the ventrolateral subregion of the PAG (vlPAG) was selected because of its role in the DPMS.^4,28^

## 2. Methods

### 2.1. Participants and ethical approval

Data were collected as part of the IMI-BioPain RCT4 trial (REC Reference 20/SW/0017), which focussed on validating the use of fMRI as a biomarker to assess analgesic efficacy in healthy human participants. Data shown here were collected from 1 site (University of Oxford) during the trial's initial screening visit, during which no analgesics were administered. All participants provided written informed consent. Inclusion criteria required that participants were healthy, right-handed (assessed by the Edinburgh Handedness Inventory, score ≥60), aged 18 to 45 years, with a body mass index between 18 and 30. Eligibility criteria excluded participants taking medications, those with chronic pain, neurological/psychiatric disorders or any clinically significant health conditions, and any MRI safety contraindications.

### 2.2. Study design

**Figure 1.**
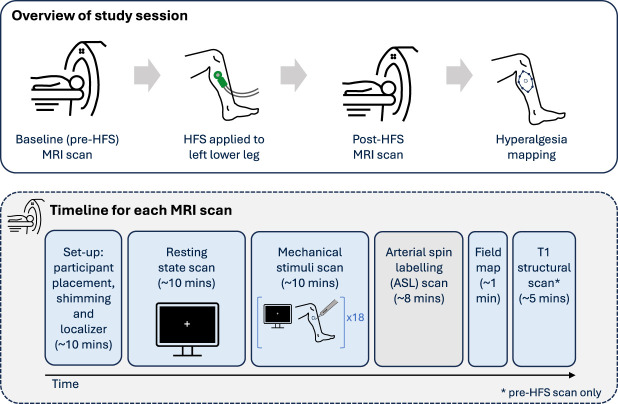
Schematic outlining the study design. There was one experimental session, during which participants completed an initial baseline (pre-HFS) MRI scan, followed by application of HFS to the left lower leg. Then, a post-HFS MRI scan was completed, followed by mapping of the secondary hyperalgesia area (top panel). The timeline for each MRI scan is outlined, which included BOLD fMRI scans during rest and during mechanical stimuli (bottom panel). HFS, high-frequency electrical stimulation; MRI, magnetic resonance imaging.

To investigate how HFS-induced CS affects brain activity at rest and in response to mechanical pinprick stimuli, we compared fMRI data collected before HFS (baseline) and approximately 20 minutes after the application of HFS, as outlined in Figure 1.

### 2.3. High-frequency stimulation

High-frequency stimulation was delivered to the skin of the medial surface of the left lower leg using the multipin HFS Electrode “EPS-P10” (MRC Systems GmbH, Heidelberg, Germany). The HFS application consisted of 5 trains of electrical pulses delivered at 100 Hz (DS7A, Digitimer, Welwyn Garden City, United Kingdom). The train duration was 1 second, with an interval of 9 seconds between each train. The stimulation intensity was set to 20x the detection threshold.^18^ After HFS application, the position of the cathode pins was marked with a pen. The electrode is designed to preferentially activate cutaneous nociceptors. Application of HFS induces a local skin flare response but does not cause long-lasting spontaneous pain.

### 2.4. Magnetic resonance imaging protocol and acquisition

Each MRI scan included a blood-oxygen-level–dependent (BOLD) scan during resting state (∼10 minutes), a BOLD scan during mechanical punctate stimulation in the secondary hyperalgesia area (∼10 minutes), an arterial spin labelling scan to measure regional blood flow during rest (∼8 minutes), and a field map (∼1 minutes). The first MRI scan (pre-HFS) also included a T1 structural scan acquired at the end (∼5 minutes). During the mechanical stimuli scan, BOLD signal changes were measured in response to 18 stimuli with a 1-second duration and an interstimulus interval of 34.5 to 38.5 seconds (jittered), applied manually using a 512-nM weighted nonskin penetrating punctate probe with a contact area diameter of 0.25 mm (MRC Systems GmbH). Each scan lasted ∼45 minutes, including set-up time to position the participant in the scanner.

Magnetic resonance imaging data were collected using a 3T Siemens PRISMA scanner with a 32-channel head-coil. All BOLD data were acquired with a whole-brain echo-planar imaging sequence with an echo time of 36 ms, field of view 192 × 192 mm, voxel size 2 × 2 × 2 mm, and multiband acceleration factor 6. The pinprick stimulation scan had 531 volumes and the rest scan had 513 volumes, both with a repetition time of 1.17 seconds. Arterial spin labelling data are not included in this report, and therefore, data acquisition is not described. A field map was acquired after the functional scans to enable correction of field inhomogeneity during analysis, with the voxel size 2 × 2 × 2 mm and field of view 192 × 192 mm. Finally, in the pre-HFS scan, the T1-weighted structural scan was acquired with a voxel size 1 × 1 × 1 mm for registration of the functional BOLD scans to standard space for group-level analysis.

### 2.5. Pain evoked by punctate stimuli

While acquiring BOLD data in the scanner, participants rated the pain intensity of each of the 18 punctate stimuli applied to variable locations within the secondary hyperalgesia area, 1 cm outside the site of the HFS cathode pins. They also provided an average rating of pain unpleasantness after all 18 stimuli. Both used a button–box interface to control a visual analogue scale (VAS) from 0 (no pain at all/not unpleasant at all) to 100 (most intense pain imaginable/extremely unpleasant).

### 2.6. Hyperalgesia mapping

Approximately 75 minutes after HFS, mechanical pinprick stimuli (512 nM) were applied in 8 radii around the cathode from the outermost point toward the centre at irregular time intervals. Participants were instructed to close their eyes and to report when/whether the stimulus intensity felt “different”—more intense, or a stronger pricking or stinging sensation, and this boundary marked. The distance between the boundary and the centre was measured. After, participants verbally rated pain intensity of the mapping stimuli on a scale from 0 (no pain at all) to 100 (most intense pain imaginable); this was one rating provided for the average pain felt at the border-defining pinprick stimuli.

### 2.7. Statistical analysis

For behavioural data, analysis was performed using GraphPad PRISM version 9.4.1 (GraphPad Software, LLC). Statistical significance threshold was *P* ≤ 0.05. Imaging data were analysed using tools in FMRIB Software Library v6.0 (FSL).^17,42,53^ For analysis of BOLD data, structural and magnitude images were brain extracted^41^ and a calibrated field map image was prepared as required for B0 unwarping. Registration to the structural image and B0 unwarping were performed using FEAT.^54^ Motion correction, spatial smoothing (5 mm for mechanical pinprick stimulation scan and 3 mm for resting state scan), and high-pass temporal filtering were applied. Independent component analysis was conducted with the MELODIC tool, and the first data sets were hand classified into signal and noise components. This training data set was then used to remove noise components from the remaining data sets using FIX.^14,38^

For the mechanical pinprick stimulation scan, once each participant's data had been denoised, an individual statistical map for the response to the pinprick stimuli was generated using the general linear model (GLM) approach implemented with FEAT.^54^ Finally, group-level whole-brain, mixed-effects analysis with a cluster-based correction for multiple comparisons was performed using FEAT to search for differences in stimulus-evoked neural activity post-HFS when compared with pre-HFS.^52^ The FSL Featquery tool was used to extract mean percentage change in BOLD parameter estimates (PEs).

For resting state analysis, the time course of BOLD activity from selected seed regions was extracted and used to generate individual statistical maps of the functionally correlated activity across the whole brain for each participant, using the GLM approach implemented with FEAT.^54^ Individual maps were constrained to grey matter only by regressing out activity in white matter and cerebrospinal fluid, using time courses for this activity in the GLM that were generated from the anatomical segmentations for each tissue type. Group-level whole-brain analysis was conducted with the same methodology applied for the pinprick data to identify differences in the seed-based Fc between the pre-HFS and post-HFS conditions.^52^ We performed 1 region-of-interest analysis to investigate whether functional connectivity between the vlPAG and posterior insular cortex (pINS) was altered, as activity in both these regions has been shown to relate to pain intensity during hyperalgesia.^30,39^ We used an anatomical mask of the pINS for the small-volume correction using nonparametric permutation testing with 5,000 permutations and threshold-free cluster enhancement, with family-wise error corrected to 0.05.^51^

### 2.8. Seed region definition

**Figure 2.**
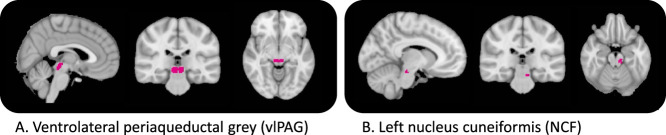
Definition of seed regions for functional connectivity analysis. Two seed regions used; ventrolateral periaqueductal grey (vlPAG—A),^8^ and nucleus cuneiformis (NCF—B), functionally defined area of increased BOLD response to mechanical stimulation induced by HFS from the whole-brain analysis, that corresponded to the NCF region previously reported.^55^ HFS, high-frequency electrical stimulation.

Selected seed regions are shown in Figure 2. The vlPAG seed region was defined based on previous studies.^8,9^ The NCF seed region was the area of significantly increased BOLD response to mechanical pinprick stimulation induced by HFS as identified from the whole-brain BOLD activation that was within the NCF region. To isolate this activity, the NCF region was defined using a 5-mm spherical mask of peak activation voxel (Montreal Neurological Institute: −10, −28, −18) identified as the NCF in a previous study of capsaicin-induced hyperalgesia.^55^

## 3. Results

Twenty healthy participants were recruited, 1 participant was excluded as they failed to meet the body mass index eligibility criteria and 1 participant did not tolerate the HFS procedure, resulting in 18 who completed the study (mean age 25.7 years, range 21–38, 9 female).

### 3.1. Pain ratings during high-frequency electrical stimulation trains

The mean intensity of the HFS stimulation was 4.26 mA ± 1.45 mA (mean ± standard deviation). The mean pain ratings (0–100, VAS) for each of the 5 trains were 49.6 ± 21.8, 57.4 ± 23.8, 60.5 ± 23.3, 62.3 ± 24.3, and 63.0 ± 23.8, respectively (mean ± standard deviation).

### 3.2. Hyperalgesia mapping results

All participants developed a discrete area of secondary hyperalgesia to mechanical stimuli with an average radius of 40.5 mm (range 12.6–72). The average pain intensity rating of the mapping stimuli (single rating provided using a VAS from 0 to 100 for the average pain felt at the border-defining pinprick stimuli) was 36.9 (range 5–75).

### 3.3. Punctate-evoked pain responses

**Figure 3.**
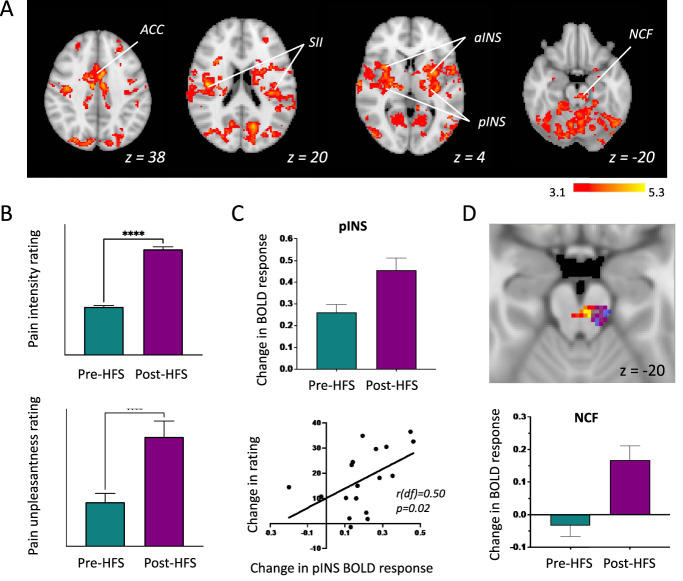
Response to punctate stimuli. (A) *Whole-brain* BOLD activation to mechanical stimulation was significantly increased during the post-HFS scan vs baseline (mixed-effects analysis, Z > 3.1, *P* < 0.05) in areas involved in pain perception such as the anterior and posterior insula cortices (aINS and pINS), anterior cingulate cortex (ACC), amygdala (AMY), hippocampus (HIP), nucleus cuneiformis (NCF), thalamus (THA), and secondary somatosensory cortex (SII). (B) Mean pain intensity (top) increased from 14.73 to 32.31 (P < 0.001, paired *t* test) and mean pain unpleasantness (bottom) increased from 16.06 to 39.78 (*P* < 0.001, paired *t* test), compared with baseline. (C) The significant increase in BOLD response in the contralateral pINS compared with baseline is illustrated in the top panel. There was a significant positive correlation between change in pain intensity ratings and change in BOLD parameter estimate for pINs between the pre-HFS and post-HFS conditions (bottom panel; Pearson correlation coefficient: r [df] = 0.50, *P* = 0.02). (D) Top image slice (MNI coordinate Z = −20): BOLD activation increases in the brainstem that includes the NCF region (whole-brain mixed-effects analysis, Z > 3.1, *P* < 0.05) is shown in red/yellow. Overlaid in blue, is a 5-mm spherical mask centered around the peak activation voxel in the NCF region in response to hyperalgesia induced by capsaicin in healthy human previously reported by Zambreanu et al. Bottom bar plot: Mean percentage parameter estimates extracted from the red/yellow area that were within the NCF region identified in blue for each condition is plotted for illustration purposes. All error bars show the SEM. HFS, high-frequency electrical stimulation; MNI, Montreal Neurological Institute.

Punctate stimuli-evoked BOLD responses and corresponding subject reported pain responses to stimuli delivered to the area of secondary hyperalgesia are shown in Figures 3A and B.

High-frequency electrical stimulation increased pain unpleasantness in all 18 individuals and intensity in 17 individuals. Post-HFS, on VAS scale from 0 to 100, the mean pain intensity (average across the 18 stimuli applied) increased significantly from 14.73 at baseline to 32.31 (*P* < 0.001, paired *t* test), and the mean pain unpleasantness ratings increased from 16.06 at baseline to 39.78 (*P* < 0.001, paired *t* test) as shown in Figure 3B.

High-frequency electrical stimulation induced a significant increase in the punctate stimuli-evoked BOLD response in areas involved in pain perception and descending pain modulation, including the pINS, mid-anterior cingulate cortex, amygdala, hippocampus, NCF, thalamus, and SII (whole-brain, mixed-effects analysis, Z > 3.1, *P* < 0.05), shown in Figure 3A.

The mean percentage change in PEs extracted from the significantly active cluster of voxels in the pINS; an area considered to play an important role in pain perception,^39^ is illustrated in Figure 3C. This increase in BOLD response in the pINS significantly positively correlated with CS-induced increase in pain intensity and unpleasantness (Fig. 3C, scatter plot).

There was an area of significantly increased BOLD activity in the brainstem NCF area. The mean percentage change in PEs extracted from within this significantly active cluster illustrates the increase in BOLD response to mechanical stimuli post-HFS (Fig. 3D, bar plot). This increase in BOLD response in the NCF did not show a significant relationship with CS-induced increase in pain intensity (r = −0.06; *P* = 0.40) and unpleasantness (r = −0.08, *P* = 0.38).

### 3.4. Resting state seed-based functional connectivity

#### 3.4.1. Ventrolateral periaqueductal grey seed region

**Figure 4.**
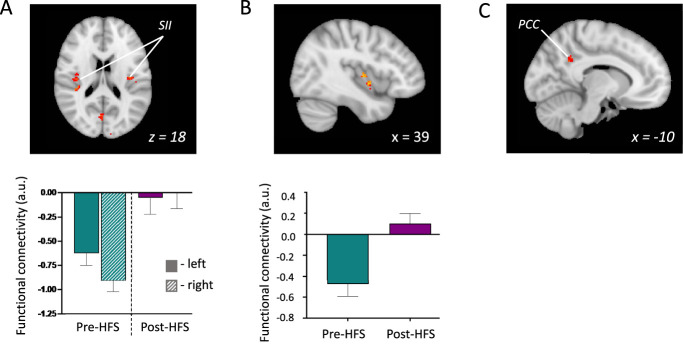
Changes in resting-state functional connectivity (Fc). (A) Whole-brain mixed-effects analysis (Z > 3.1, *P* < 0.05) exploring seed-based Fc with the ventrolateral periaqueductal grey (vlPAG) seed region showed Fc significantly increased between the vlPAG and the secondary somatosensory cortex (SII) compared with baseline (top panel). Fc coefficients extracted from the anatomical left and right SII regions show that this “increase” reflects a change from negative Fc to a less negative value (close to zero) from the pre-HFS scan to the post-HFS scan (bottom panel). (B) Region-of-interest (ROI) analysis of the right posterior insula (pINS) with the vlPAG seed region showed increased Fc between these areas compared with baseline (top panel; evaluated with a small-volume correction using nonparametric permutation testing with 5,000 permutations and threshold-free cluster enhancement, with family-wise error corrected to 0.05). Extracted Fc coefficients show that this reflects a switch from negative connectivity to positive connectivity (bottom panel). (C) Whole-brain resting seed-based functional connectivity during hyperalgesia (post-HFS) compared with baseline for the NCF seed region showed there was significantly decreased connectivity in the posterior cingulate cortex (PCC) region (mixed-effects analysis, Z > 3.1, *P* < 0.05). MNI-512 coordinates are shown, and all error bars show the SEM. HFS, high-frequency electrical stimulation; MNI, Montreal Neurological Institute; NCF, nucleus cuneiformis.

Functional connectivity was significantly increased between the vlPAG seed region and clusters corresponding to the right thalamus and the bilateral secondary SII (whole-brain mixed-effects analysis, Z > 3.1, *P* < 0.05) (Fig. 4A—top image slice Montreal Neurological Institute coordinate z = 18). There were no areas of decreased connectivity. Plotted Fc coefficients extracted from these areas of activation within the left and right SII regions illustrate that this “increase” reflects a change from negative Fc to a less negative value (close to zero) from the pre-HFS scan to the post-HFS scan, as shown in Figure 4A bar plot. The SII region was anatomically identified using the Juelich Histological Atlas in FSL.^6,7^

Capsaicin-induced CS was previously shown to modulate PAG and pINs activity.^30,39^ We performed a region-of-interest analysis using small-volume correction to interrogate the connectivity between the vlPAG and the right pINS. There was a significant HFS-induced increase in Fc between the vlPAG and pINS, from −0.48 pre-HFS to 0.10 post-HFS, shown in Figure 4B. Extracted Fc coefficients show that this increase is caused by a change from negative connectivity to positive connectivity from the pre-HFS condition to the post-HFS condition. This change in Fc did not show a statistically significant correlation with change in pain intensity (r = −0.27, *P* = 0.14) or unpleasantness (r = −0.36, *P* = 0.07).

#### 3.4.2. Nucleus cuneiformis seed region

Whole-brain seed-based Fc analysis using the NCF as the seed region showed that Fc decreased between the NCF and the posterior cingulate cortex (PCC) in the post-HFS condition compared with the pre-HFS condition (whole-brain mixed-effects analysis, Z > 3.1, *P* < 0.05), as shown in Figure 4C. There were no areas of increased connectivity.

## 4. Discussion

The use of fMRI in conjunction with experimental models of CS in healthy humans has proven highly valuable for the investigation of the brain neural correlates of CS and its modulation by analgesics—as potential experimental medicine biomarkers for early drug discovery.^16,24,25,49,55^ Using neuroimaging, modulation of activity in cortical regions such as the insular cortex, ACC, thalamus, and SII has been reported consistently in other experimental models of CS. High-frequency electrical stimulation is an established human model of CS. However, information relating to its neural correlates is sparse and it had not previously been studied in conjunction with fMRI. In this study, HFS successfully induced an area of secondary mechanical hyperalgesia in a cohort of healthy participants, as demonstrated with significant increases in mean pain intensity and unpleasantness ratings. Using fMRI, we showed increased neural activity in cortical and subcortical pain processing areas and key brainstem nuclei such as the NCF in response to mechanical pinprick stimuli consistent with findings from human experimental models of capsaicin-induced CS. Furthermore, we showed that Fc between brain regions is altered during rest after induction of a state of CS by HFS. Although potentially limited by a relatively small sample size, these findings show that CS induced by HFS initiates changes in top-down as well as bottom-up controls of pain sensitivity, thus indicating that use of the HFS model is relevant to further our understanding of pain chronicity and susceptibility. This is aligned with a recent study which suggests that preoperative susceptibility to develop HFS-induced secondary hyperalgesia is associated with post-thoracotomy pain at 2 months, although this was conducted in a small number of patients.^13^

### 4.1. Evoked neural activity

Modulation of activity in cortical regions implicated in pain perception, such as the insular cortex, ACC, thalamus, and SII has been reported consistently in imaging studies using other experimental models of CS^25,29,37,55^ and is consistent with the results of our study (Fig. 2A).

The pINS is an area of particular interest for human pain perception as an early processing site in the lateral nociceptive system for nociceptive signals.^1,10,11,26^ Furthermore, evoked neural activity in the pINS during a capsaicin-induced state of CS in healthy participants has been shown to be differentially modulated by analgesics.^49^ Cerebral blood flow in this area increases in response to tonic pain induced by capsaicin and is strongly correlated to pain intensity ratings.^39^ Consistent with these findings, our study demonstrated increased neural activity in pINs induced by the application of HFS and that this activity positively correlated with the increase in pain ratings (Fig. 2C). This underlines that the (unchanged) peripheral input initiated by pinprick stimuli that is enhanced by sensitisation of spinal dorsal horn neurons indeed leads to enhanced responses in the thalamo-cortical parts of the nociceptive system.

Two studies that aimed to characterise the neural correlates of CS using capsaicin models (topical capsaicin and intradermal capsaicin) reported increased activity in an area of the brainstem in response to nociceptive stimulation consistent with the location of the NCF.^25,55^ This result has been replicated in this study with the HFS model, where there is a significant increase in NCF activation after HFS compared with baseline, again in response to nociceptive stimulation. Interestingly, unlike the pINs region, this increased activity in the NCF region did not track the increased hyperalgesic pain intensity perception, similar to the finding from a previous study of capsaicin-induced CS.^25^ This is in line with the concept that the brainstem activity is more related to the spinal dorsal horn facilitation induced by the peripheral agent rather than the perception of hyperalgesia per se. Preclinical studies provide ample evidence for the role of the brainstem nuclei in facilitation of nociceptive inputs and the maintenance of CS.^43,45,50^ The resolution (2 × 2 × 2 mm) and smoothing (5 mm) applied can limit spatial specificity for small brainstem regions such as the NCF, and future studies are recommended to improve localisation with brainstem-optimised protocols.

### 4.2. Resting connectivity in a centrally sensitised state

During rest, seed-based Fc analysis demonstrated an increase in connectivity between the vlPAG, a key component of the DPMS, and the bilateral SII and right pINS cortex, both regions involved in pain processing. The vlPAG seed region showed negative Fc with the bilateral SII and right pINS cortex regions in the pre-HFS baseline condition, which switched to a close to zero or positive connectivity in the post-HFS condition. Previously, negative connectivity has been less well-studied and interpretation of such findings is debated, but evidence is growing that it likely does have a physiological basis.^31^ Negative Fc has been previously shown between the PAG (a key region involved in pain modulation) and the pINS cortex during rest.^21^ The switch from negative connectivity (during rest) to positive connectivity (during hyperalgesia) may reflect modulation of pain responses during the hyperalgesic state mediated by the PAG. The vlPAG, through its role in descending pain control, may be exerting an inhibitory influence on the SII and pINS under normal conditions, with the shift from negative connectivity to zero connectivity in the sensitised state indicating a disruption of the normal inhibitory control with a shift toward more facilitation. This is consistent with recent work indicating that after HFS, excitatory signalling is no longer controlled by inhibitory mechanisms.^34^

### 4.3. High-frequency electrical stimulation model advantages and disadvantages

Overall, the HFS model has several characteristics which make it a valuable tool for use in conjunction with neuroimaging. It offers a long and stable duration of CS,^15,18,35^ which is advantageous in imaging studies enabling extended data acquisition within a single session or across multiple time points in longitudinal designs. The rapid and consistent onset of CS, peaking at around 30 minutes, enables the model to be used for assessing the efficacy of interventions administered after the establishment of the sensitised state (post-HFS), more closely mimicking the scenario for treating patients who already have developed chronic pain. In previous studies using capsaicin models, analgesics are often given before the model set-up,^16,49^ confounding interpretation of whether the intervention is modulating the establishment or maintenance of CS. The HFS model lends itself more to a standardized implementation in clinical trials than the intradermal capsaicin model that is very much operator-dependent. Compared with the topical capsaicin model, the HFS model allows CS effects to be studied in the absence of ongoing pain, which is also an advantage over the continuous low frequency stimulation (LFS) model.^22,36^

Another feature of HFS, consistent with our results, is the high reported response rates of 79% to 100%.^2,19,20,35,36^ This is advantageous as it maximises the effect size within the data set and reduces the likelihood of excluding participants due to insufficient hyperalgesia, an important consideration for resource-intensive imaging studies. One disadvantage of the HFS model is that due to the painful nature of the stimulation, it is challenging to include a blinded sham session. Lack of blinding or randomisation was a limitation of this study, and therefore, we cannot be certain that changes in pinprick-evoked responses and resting state Fc are not also related to a general effect of time, or what was experienced during HFS (independently of HFS-induced secondary hyperalgesia). A blinded variation in frequency of stimulation could have been considered for a control, but application of electrical low-frequency stimulation induces spinal LTD and decreased ratings of subsequent electrical stimuli, but also hyperalgesia to pinprick^18^; hence, this would be unlikely to yield different outcomes. Alternatively, data could have been collected during pinprick stimulation of the sensitised site and a nonsensitised control site, but this would not have provided a control for the resting state condition. As we observed changes in Fc during rest, which may result in altered processing of any nociceptive inputs, this may have resulted in changes in responses to pinprick in the nonsensitised control site too, compared with baseline.

## 5. Conclusion

This study has shown that the neural correlates of HFS-induced CS, as measured with fMRI, include increased activation of pain-related brain areas as well as regions involved in the descending pain control system, with changes in Fc suggesting a shift in descending controls toward facilitation. High-frequency electrical stimulation has promising characteristics that make it a valuable tool for use in future studies. Advantages and potential disadvantages associated with the HFS model need to be carefully considered when designing experiments that use this tool, as with any other experimental model applied in a research setting.

## Disclosures

S.C., V.W., R.R., O.C., A.M., F.F., R.D.T., and I.T. declare no conflicts of interest relating to this work. N.B.F. has received consultancy fees from PharmNovo, NeuroPN, Saniona, Nanobiotix, and Neurvati, and has undertaken consultancy work for Aarhus University with remunerated work for AKIGAI, Biogen, Merz, and Confo Therapeutics, outside the submitted work.
